# Structural analysis of glycoproteins: building N-linked glycans with *Coot*

**DOI:** 10.1107/S2059798318005119

**Published:** 2018-04-06

**Authors:** Paul Emsley, Max Crispin

**Affiliations:** a MRC Laboratory of Molecular Biology, Francis Crick Avenue, Cambridge Biomedical Campus, Cambridge CB2 0QH, England; b Centre for Biological Sciences and the Institute for Life Sciences, University of Southampton, Southampton SO17 1BJ, England

**Keywords:** *Coot*, X-ray model building, N-linked glycans, glycoproteins

## Abstract

*Coot* is a graphics application that is used to build or manipulate macromolecular models; its particular forte is manipulation of the model at the residue level. The model-building tools of *Coot* have been combined and extended to assist or automate the building of N-linked glycans. The model is built by the addition of monosaccharides, placed by variation of internal coordinates. The subsequent model is refined by real-space refinement, which is stabilized with modified and additional restraints. It is hoped that these enhanced building tools will help to reduce building errors of N-linked glycans and improve our knowledge of the structures of glycoproteins.

## Abstract 2

Newly improved tools for carbohydrate modelling in *Coot* are presented.

## 1. Introduction   

Cell-surface and secreted proteins are often modified by numerous asparagine (N)-linked glycans. In addition to their role in lectin-mediated protein folding, glycans often play a structural role by forming intramolecular interactions with the protein surface which can stabilize protein domains and influence dynamics (Petrescu *et al.*, 2006 ▸). Although glycans have the capacity to be highly dynamic and therefore conformationally heterogenous, they are increasingly being observed by both X-ray crystallography and cryo-electron microscopy (cryo-EM; see, for example, Bai *et al.*, 2015 ▸). This trend includes a growing number of examples of glycans that are braced against protein surfaces, including by antibody binding (Pejchal *et al.*, 2011 ▸), and by the advent of methods to manufacture chemically homogenous glycoforms for structural analysis (Chang *et al.*, 2007 ▸).

The modelling of carbohydrates using X-ray data has long been problematic and has not been well supported in macromolecular-modelling tools (Crispin *et al.*, 2007 ▸; Agirre, Davies *et al.*, 2015 ▸). In recognition of these limitations, several tools have been developed to validate models of glycans, including *CARP* (Lütteke *et al.*, 2005 ▸), *pdb-care* (Lütteke & von der Lieth, 2004 ▸) and *Privateer* (Agirre, Iglesias-Fernández *et al.*, 2015 ▸). These provide insights into the monosaccharide connectivity and orientation. In particular, detailed analysis of the torsion angles between monosaccharides and internal pyranose-ring conformations can be generated to identify potentially incorrect structures (Joosten & Lütteke, 2017 ▸).

Although Agirre (2017 ▸) has recently described the structural principles that should be adopted for accurate model building of glycans, it is recognized that in practice it is not straightforward to reliably adhere to these ideals given the limited functionality of current building tools, including the carbohydrate module hitherto available in *Coot* (Agirre *et al.*, 2017 ▸). Here, the general-purpose nature of the building and refinement tool available in *Coot* (Emsley *et al.*, 2010 ▸) is exploited to provide a richer environment for the accurate building of N-linked glycans. We present multiple build environments allowing the user to select automated glycan building, guided model building (where the build options are shaped by the expected glycan structure) and a manual build option (where the user can direct the monosaccharide and linkage type). With the growing range and sophistication of biophysical data describing glycoprotein structures, we hope that the presented advances in building tools will enhance our understanding of this important class of biomolecules.

## 2. Method   

We wanted to provide a tool in *Coot* that was interactive and could provide the user with a knowledge-based model-building guide through glycan space. The carbohydrate-building tool was designed to have three modes.

(i) ‘Expert User’ mode: monomer-by-monomer addition of the ‘next’ monosaccharide. The user chooses the link type and the monosaccharide type. The different hypotheses for the position, orientation and conformation of the ‘next’ monosaccharide are assessed and the best one is added and refined; control is then returned back to the user.
(ii) Linked Monosaccharide Addition (LMA): as above, with the modification that *Coot* uses glycan comprehension. Given a (user-selected) glycan type, only certain monosaccharide types with certain link types are available for any given position on the tree [for example, only *N*-acetyl-β-d-glucosamine (NAG) linked by ‘NAG-ASN’ is available for the first position].
(iii) Whole Tree Addition (WTA), where the user need only identify the starting asparagine and the glycosylation tree type to be added. The options are ‘High Mannose’, ‘Hybrid (Mammal)’, ‘Complex (Mammal)’ and ‘Complex (Plant)’. This mode automatically (*i.e.* without user intervention) applies built-in knowledge of residues and link types for particular glycosylation trees, and uses density fit for branch termination.

### 2.1. Coordinate generation   

Building new saccharide residues in *Coot* involves the initial assignment of atom positions and temperature factors (also known as *B* factors), followed by subsequent refinement which respects stereochemical principles.

#### 2.1.1. Temperature factors   

Because there is no temperature-factor refinement of atoms in *Coot*, the temperature-factor model for added carbohydrate atoms is necessarily crude. The atoms of the generated monomers are given a temperature factor of 1.55 times the median of the atoms in the environment (*i.e.* atoms of residues within 5 Å of the glycan) of the glycosyl­ation [this being the factor by which the median temperature factors of the atoms of N-linked glycans in the wwPDB archive (Berman *et al.*, 2003 ▸) are greater than those of their environment].

#### 2.1.2. Torsion-angle variation   

The creation of models based on torsion-angle variation (obviously) depends on the identification of torsionable bonds (pyranose-ring torsions are not used in torsion-angle hypothesis generation). Torsion bonds, including those torsion bonds that result from the glycosidic linkage, are derived from the *REFMAC* monomer library (Vagin *et al.*, 2004 ▸).

#### 2.1.3. Atom positions   

A stochastic hill-climbing algorithm with simulated annealing is used for hypothesis generation of the position and conformation of the isomer of the added pyranose by variation of the linking φ and ψ torsion angles and the internal χ angles. The degree of variation (that is to say the width of the probability distribution) of the torsion angles both within any one conformer and the glycosidic bond conformation decreases with increasing cycle number. (For the sake of clarity, the variation within a conformer might be ∼10° and that between conformers might be ∼120°.)

The crystal structure of an α-bungarotoxin complex (PDB entry 2qc1; Dellisanti *et al.*, 2007 ▸) was used, after model idealization, as a reference to determine the template internal coordinates (in particular the torsion angles) for *N*-acetyl-β-d-glucosamine (NAG), α-d-mannose (MAN) and β-d-mannose (BMA).

The model idealization was performed on the glycan attached to residue 141B using *Coot*’s regularize-residue function with the *REFMAC* monomer library, including torsion restraints.

Unsurprisingly, not all torsion-angle variants have an equal probability of being close to the true solution, and it is quite possible that the initial unfitted model itself (generated simply from starting coordinates merely orientated relative to the underlying target residue or asparagine) can provide a hypothesis that is quite close to the true solution. In such cases, the best solutions would be found by only small variations of the torsion angles (that is, without the exploration of alternative conformers or glycosidic bond conformers). Therefore, the first 15% of trials are generated in this mode (with conformer and glycosidic bond conformer variation turned off) and the model can be optimized with local hill-climbing before comparison with conformer and glycosidic bond conformer alternatives.

#### 2.1.4. Hypothesis testing   

The fit to density is assessed by the sum of the atomic-weighted density values of the hypothesis of the residue non-H-atom positions. If the fit of the hypothesis is better than the current fit, then the hypothesis atom positions are used to replace those of the current best fit and are then used for future rounds of torsion-angle variation.

### 2.2. Refinement   

*Coot*’s real-space refinement is used to refine the selected residues. The selected residues are typically the residue at the centre of the screen and the residues to which it is covalently bonded.

Monosaccharide dictionaries generated from *AceDRG* (Long *et al.*, 2017 ▸) are used in preference to those currently in the *REFMAC* monomer library (which *Coot* would otherwise use by default). These *AceDRG*-derived dictionaries are an improvement over previous dictionaries in the *REFMAC* monomer library (Agirre, 2017 ▸).

Real-space refinement of the selected monosaccharides is stablilized by the use of aperiodic torsion-angle restraints [the target torsion angles are copied from the dictionary output of *Privateer*, which in turn is generated from the ideal models in the Chemical Component Dictionaries (Westbrook *et al.*, 2015 ▸) for the various pyranoses, which in turn are generated by the *OpenEye* software (Boström *et al.*, 2003 ▸)].

*ProSMART* (Nicholls *et al.*, 2014 ▸) is often used to generate local distance restraints based on a high-resolution reference structure to stabilize the *REFMAC* refinement of a lower resolution structure (Nicholls *et al.*, 2012 ▸). In so doing, the target function for any particular distance is not that of a typical harmonic distance restraint, but is modified by a Geman–McClure M-estimator, so that the target function and gradient for distances between atom pairs that are far from the target value are relatively lessened. Such distances and target functions have been re-purposed so that a consensus model derived from carbohydrate models in crystal structures deposited in the wwPDB can be used to stabilize the real-space refinement in *Coot*.

#### 2.2.1. Generation of external distance restraints   

The structures in the wwPDB archive were searched for N-linked glycans. Structures proceeded to the statistics-generation step if they passed the following criteria.

(i) The tree should contain at least Asn-NAG-NAG-BMA.
(ii) Structure-factor data were available.
(iii) The ‘status’ of the monomers as identified by *Privateer* must be marked as ‘OK’.

The interatomic distances of every non-H atom of every residue-pair type [where a residue-pair type identifies a residue by its branch number, residue type, link type and parent residue type and might be, for example, 2: NAG-β(1–4)-NAG] were enumerated. The statistics of each interatomic distance type were calculated, including the mean, median and an indicator of multi-modality: the modified Sarle coefficient (Long *et al.*, 2017 ▸).

#### 2.2.2. Use of external distance restraints   

External distance restraints were used for linked residue atom pairs if there were at least 20 examples and the modified Sarle coefficient was less than 0.42. The Geman–McClure α value used in the real-space refinement was set at 4.2.

### 2.3. Whole Tree Addition exclusion criteria   

In WTA mode, *Coot* needs to decide whether the most recently added monomer in the current model is of sufficient quality to try to continue adding residues along that branch. This is assessed using the fit to density, *i.e.* the density correlation coefficient between the model and the map (the 2*mF*_o_ − *DF*_c_ map as output by *REFMAC*). If the correlation coefficient is below 50% (the default value) then this residue is removed and building along that branch is terminated. It should be noted that Agirre, Davies *et al.* (2015 ▸) have found that the correlation coefficient is often higher than 50% if the model is allowed to distort during refinement.

### 2.4. Test-data set   

All 23 structures/data sets for N-linked glycans consisting of at least β-mannosylated *N*,*N*′-diacetylchitobiose (ASN-NAG-NAG-BMA; ManGlcNAc_2_) uniquely published and deposited in the wwPDB from Jan 2017 to June 2017 (inclusive) for which structure-factor data were available were used to test the new building tools (if multiple structures were reported in the same article, then only the first structure was used for testing). The structures used in the test data set are PDB entries 5mwf (Suckling *et al.*, 2017 ▸), 5mx0 (Paracuellos *et al.*, 2017 ▸), 5mya (Leppänen *et al.*, 2017 ▸), 5ug0 (Liu *et al.*, 2017 ▸), 5ugy (Whittle *et al.*, 2011 ▸), 5um8 (Guenaga *et al.*, 2017 ▸), 5wzy (Kasuya *et al.*, 2017 ▸), 5n09 (Rouvinski *et al.*, 2017 ▸), 5n11 (Bakkers *et al.*, 2017 ▸), 5uqy (Hashiguchi *et al.*, 2015 ▸), 5utf (Chuang *et al.*, 2017 ▸), 5x2p (Nuemket *et al.*, 2017 ▸), 5v2a (Thornburg *et al.*, 2016 ▸), 5v4e (Lee *et al.*, 2017 ▸), 5v7j (Zhou *et al.*, 2017 ▸), 5vaa (Labrijn *et al.*, 2017 ▸), 5vgj (Cale *et al.*, 2017 ▸), 5vh5 (Lerch *et al.*, 2017 ▸), 5vk2 (Hastie *et al.*, 2017 ▸), 5nuz (Zeltina *et al.*, 2017 ▸), 5nxb (Hill *et al.*, 2017 ▸), 5o32 (Xue *et al.*, 2017 ▸) and 5vtq (Wu *et al.*, 2017 ▸).

The maps for each structure were generated using the MTZ files available from the Electron Density Server (Kleywegt *et al.*, 2004 ▸) at PDBe. The test data sets were used in both the Linked Monomer Addition mode and the Whole Tree Addition mode.

### 2.5. Validation software   

*Privateer* was used for the validation of all carbohydrate models. Unfortunately, the output files created by *Coot* could not be parsed by *pdb-care* from glycosciences.de (Lütteke & von der Lieth, 2004 ▸) so this could not be used for additional validation.

### 2.6. User interaction   

**Figure 1:**
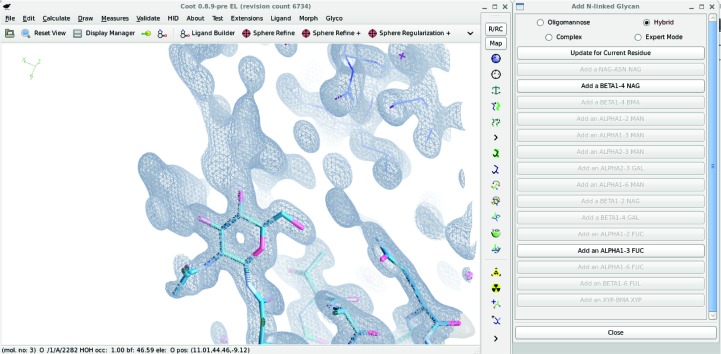
The N-linked glycan builder Linked Monomer Addition mode in action. The dialogue is aware of the current ‘active’ residue and its place in the glycosylation tree and modifies the ‘sensitive’ state (*i.e.* the ability to be responsive to clicks) of the buttons accordingly. In this case, the inital asparagine-linked NAG has been placed and the dialogue invites the user to add a β(1–4)-linked NAG or an α(1–3)-linked fucose (both of which have plausible-looking density).

This tool is activated in *Coot* using Extensions → Modules → Carbohydrate, which provides a menu called ‘Glyco’ with carbohydrate tools. The Whole Tree Addition mode is activated by choosing ‘High Mannose’, ‘Hybrid (Mammal)’ (*etc.*) from the ‘Glyco’ menu. The Linked Monomer Addition mode is activated by clicking the ‘N-linked dialog’ menu item (Fig. 1 ▸). This provides a dialogue window that is aware of the position of the active residue in the glycan tree structure and changes the buttons for the next monomer addition accordingly. This interface is available in the 0.8.9 release.

## 3. Results   

### 3.1. Linked Monomer Addition   

**Figure 2:**
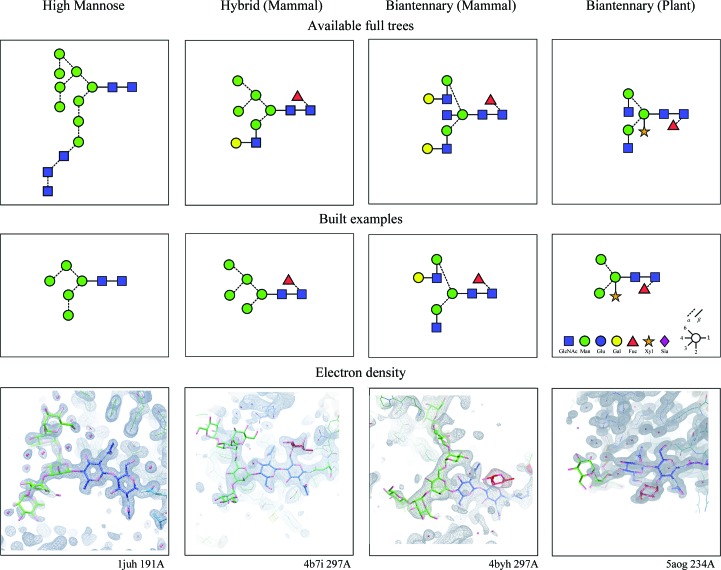
Comprehension of N-linked glycosylation trees that has been built into *Coot*. For ease of use, the trees are partitioned into five major types (four of which are shown). The user decides which tree type is to be built, and only the given linked residue types are then available for any position in the tree. The rationale for these categories is the biochemical distinction of the major types of glycan structures that could arise from the common expression systems used to generate structures deposited in the PDB. In addition, some finer distinctions are made (for example, between plant and mammalian variants of complex and hybrid-type glycans) to help the user avoid or accommodate species-specific differences. In the case of plants, these include the specific α1–3-fucose off the asparagine-linked NAG and β-xylose linked to the β-d-mannose (BMA) (Schoberer & Strasser, 2017 ▸). The aim is to help to reduce errors when users are less familiar with the residues and linkages that should be expected for particular types of glycan (Crispin *et al.*, 2007 ▸). The top row represents the full tree that is available for a given tree mode. [Unfortunately, at the present time, the addition of sialic acid to the galactose in the ‘Biantennary (Mammal)’ and ‘Hybrid (Mammal)’ trees is not available owing to unresolved compatibility problems with the dictionary linking information.] Using the LMA mode, *Coot* has been used to build representative examples for each tree type. The second row shows the cartoon for the built tree using the nomenclature of the Consortium for Functional Glycomics (CFG) with link sensitivity. The third row shows the electron density represented by the cartoon above. The carbon atoms of the individual monosaccharides are coloured using the CFG convention.

Fig. 2 ▸ shows the built-in N-linked tree comprehension. The LMA mode was used (with little effort) to build example glycan extended trees for four example structures (with better than average density for the carbohydrate).

The results of the glycan model building using the LMA mode are shown in Table 1 ▸. It was straightforward in most cases to recapitulate the tree structures in the LMA mode. In many cases the LMA models closely matched those of the deposited structures.

The correlation coefficients of the LMA model are routine­ly lower than those of the deposited structures. The atoms were in different positions, but most of the difference is probably owing to the lack of temperature-factor refinement of the LMA model. It is important to note that the correlation coefficient was not used as a criterion for branch termination or quality of fit. Instead, model quality was examined by eye if needed; however, in most cases tree termination was decided based on the lack of density for the next monomer.

All carbohydrate models built in the LMA mode were marked ‘OK’ by *Privateer*.

### 3.2. Whole Tree Addition   

This (automated) mode less frequently recapitulated the deposited structures. This mode often (about 50% of the time) created a model that contained fewer monosaccharides than the deposited structure. Again, the correlation coefficients of the WTA models were lower than those of the deposited models.

All carbohydrate models built in the WTA mode were marked ‘OK’ by *Privateer*.

### 3.3. Cryo-EM reconstructions   

While the main target of this tool was use with crystallo­graphic data, it was also tested with a few cryo-EM reconstructions: PDB entries 5xsy (Yan *et al.*, 2017 ▸), 5x0m (Shen *et al.*, 2017 ▸) and 5vn8 (Ozorowski *et al.*, 2017 ▸). This tool did not work well with these maps. Firstly, the module naively set the weight to a ‘tight’ value that worked for the tested maps generated from X-ray data (which were more or less on the absolute scale) but is wrong for cryo-EM reconstructions. If this was fixed manually then a second problem became apparent. The cryo-EM reconstructions tested were of noticably lower resolution than the X-ray maps tested. The maps have little to no density for the *N*-acetyl group of the NAGs, and trying to fit this pushes the model over, which means that the next NAG is misplaced and the real-space refinement cannot recover the correct orientation. It may be possible to address this second issue, but it does not seem straightforward to do so.

## 4. Discussion   

### 4.1. Model-building tools in *Coot*   

By using the extant model-building tools in *Coot* and adding comprehension of carbohydrate chemistry, we have created a tool that can add N-linked carbohydrates to protein models without nomenclature errors and that, in the LMA mode, can create a model that matches that which an expert would build, with little effort.

Using the WTA mode with better than average resolution maps (for example those used for Fig. 1 ▸), *Coot* builds carbohydrate models that closely match the deposited model. For the given 2017 test structures, however, in several cases the WTA mode often failed to recapitulate the reference structure when the resolution limit of the data was poorer than average. When the WTA model is annotated as ‘more’ it seemed to us that there was good reason to extend the model in the way that the WTA mode had done. In two cases the WTA mode added a monomer that was probably (but not unequivocally) wrong.

In future, temperature-factor refinement (for example, using the shift-field refinement of isotropic displacement factors; Cowtan & Agirre, 2018 ▸) and possibly other exclusion criteria will improve the accuracy of the correlation coefficient and thus the accuracy of new monosaccharide rejection.

### 4.2. Extension to O-linked glycans   

O-linked glycans were not part of this investigation. In order to support O-linked glycans, the consensus distances will need to be determined, where more care may need be taken in their selection and weighting because there are fewer models to provide distances. The infrastructure is in place to handle them when this has been performed.

### 4.3. Interpretation of structural data for glycans   

While much structural interpretation can be made using crystallographic or microscopic data alone, further knowledge of the underlying chemical compositions of glycans is an important guide in the building of accurate models. This knowledge can be derived from (i) a general understanding of the range of glycans that can be expected to occur from a particular expression system or biological source (see, for example, Davis & Crispin, 2010 ▸); (ii) deliberate manipulation of the glycosyation pathway either during expression (see, for example, Crispin *et al.* (2009 ▸) or by *in vitro* enzymatic manipulation (see, for example, Krapp *et al.*, 2003 ▸; Crispin *et al.*, 2013 ▸); or (iii) analytical characterization of the glycans or glycopeptides. In practice, investigators focusing on glycosylation often use multiple factors to inform building. However, electron density for glycans can also arise in the course of a project where the user has little prior expectation of glycan compositions.

Despite significant variation in the chemical heterogeneity of glycosylation across different expression systems, the glycan pathway shows significant conservation in the endoplasmic reticulum and only shows significant divergence in the spectrum of glycosyltransferases that are present in the Golgi apparatus. One important consequence of this is that glycans that form extensive interactions with protein surfaces are often trapped as high-mannose-type glycans (Man_5–9_GlcNAc_2_) regardless of the capacity of the producer cell for complex-type glycosylation (Crispin *et al.*, 2004 ▸; Loke *et al.*, 2016 ▸). In addition to glycan–protein interactions limiting α-manno­sidase processing, glycan–glycan clustering can also lead to the ectopic secretion of high-mannose glycans (Pritchard *et al.*, 2015 ▸).

As X-ray crystallography requires restricted conformational variation to give interpretable electron density, it is often the sterically restricted high-mannose glycans that give interpretable electron density (Davis & Crispin, 2010 ▸). In other examples, protein–glycan interactions can stabilize and limit the heterogeneity of complex-type structures. In the homodimeric IgG Fc domain, a core fucosylated and partially galactosylated biantennary glycan extends across the surface of the Cγ2 domain, giving rise to extended interpretable electron density. The stabilizing environment of the Fc glycans also means that engineered Fc glycoforms containing oligomannose-type or hybrid-type glycans also exhibit ordered scattering across almost the entire glycans (Bowden *et al.*, 2012 ▸; Crispin *et al.*, 2009 ▸).

Glycan engineering to homogenize the chemical heterogeneity of glycoproteins has been used to enable complete deglycosylation using endoglycosidases (Chang *et al.*, 2007 ▸). While this has aided the crystallization of an extensive range of glycoproteins, it has increasingly been noted that the deglycosylation of such homogenous glycoforms is not always necessary for crystallization (Bowden *et al.*, 2009 ▸; Stewart-Jones *et al.*, 2016 ▸). However, artificial restriction of the glycan heterogeneity is usually an important aid to crystallization. For example, the glycans can be trapped as Man_9_GlcNAc_2_ using the α-mannosidase inhibitor kifunensine (Chang *et al.*, 2007 ▸). Similarly, cell lines with naturally restricted diversity can be used, such as the *Drosophila melanogaster* SC2 or baculovirus/*Spodoptera frugiperda* Sf9 systems, in which the glycans are dominated by a fucosylated derivative of the paucimannose structure (Man_3_GlcNAc_2_; Zajonc *et al.*, 2005 ▸).

Analytical characterization of the glycans can often help to support the interpretation of structural data. For example, glycan analysis has supported the building of a weakly scattering α(2–6)-linked sialic acid residue presented on a bi­antennary complex-type glycan (Crispin *et al.*, 2013 ▸). However, ambiguities can still arise. Gristick *et al.* (2016 ▸) derived glycan structures of a recombinant mimic of the HIV virion spike using crystallographic diffraction data alone, which they acknowledged to deviate from the predominant structures derived by mass spectrometry (Behrens *et al.*, 2016 ▸). This underscores the difficulty that can arise in interpreting the structural signal for a glycan, which actually represents an average signal from many molecules. Furthermore, this also underscores the possibility of the selective crystallization of glycoforms from within a heterogenous glycoprotein sample.

We envisage an increasing need for careful intepretation of glycan structural data as glycans are increasingly observed by cryo-EM, where there is no requirement for lattice contacts and no steps need to be taken to reduce the chemical or the structural heterogeneity of glycosylation (Lyumkis *et al.*, 2013 ▸; Lee *et al.*, 2015 ▸).

## 5. Summary   

The work described here is motivated to help to tackle the challenge of accurately interpreting both crystallographic and cryo-EM maps of glycans and in part to address the concerns raised by Agirre *et al.* (2017 ▸). The LMA mode is the mode that we imagine that users will find most useful.

The automated WTA mode can be expected to work with a data resolution better than 2 Å but, as the results show, at lower resolutions it cannot be relied on to make the same judgement calls that an experienced user would make.

The correlation coefficient limit is the main determinant of whether a monosaccharide is added to the model in the WTA mode. It is a user-settable parameter and can be made more permissive.

It seems likely that the tree-building would be enhanced by substructure temperature-factor refinement (sufficiently fast for interactive building).

## Supplementary Material

Coot carbohydrate fitting test data.. DOI: 10.1107/S2059798318005119/ba5284sup1.tgz

## Figures and Tables

**Table 1:** Builder results ‘Code’ is the wwPDB accession code. ‘Resolution’ is the nominal resolution limit of the data (in Å). ‘Max level’ is the maximum branching level of the reference tree. ‘Residue’ is the residue number and chain identity of the asparagine onto which the glycosylation tree was built. ‘Ref CC’ is the correlation coefficient of the glycosylation tree (including the asparagine) of the deposited reference structure to the map. ‘LMA CC’ and ‘WTA CC’ are the correlation coefficients of the glycosylation tree (including the asparagine) of the models built in LMA mode and WTA mode, respectively, to the map. ‘LMA match’ and ‘WTA match’ compare the built trees with the reference tree of the deposited structure: this is a comparison of the residues and links of the tree and not an assessment of the differences of the atom positions in the models. The ‘Notes’ column is used to remark on the performance of the WTA mode. ‘+’ means that the WTA mode added additional model, and ‘−’ means that the model from the WTA mode lacked part of the model when compared with the deposited structure. Additionally, ‘− BMA +’ means that the WTA mode model lacked the β-linked mannose and everything beyond it.

Code	Resolution	Max level	Residue	Ref CC	LMA CC	LMA match	WTA CC	WTA match	Note
5mwf	2.80	3	153A	0.852	0.744	More	0.745	More	+ α(1–6)-MAN†
5mx0	2.21	3	127A	0.800	0.715	Match	0.697	Match	
5mya	2.90	4	560A	0.834	0.719	Match	0.733	Match	
5ug0	3.40	3	91A	0.803	0.750	Match	0.714	More	+ α(1–6)-MAN
5ugy	2.80	3	91A	0.784	0.727	Match	0.622	More	+ α(1–3)-MAN
5um8	3.95	4	156G	0.732	0.740	Match	0.725	More	+ implausible 5′ MAN
5wzy	2.80	5	213A	0.848	0.810	Match	0.761	More	+ α(1–6)-MAN
5n09	3.90	4	153A	0.780	0.631	Match	0.656	Less	− BMA, α(1–3)-MAN, α(1–6)-MAN
5n11	2.45	5	315A	0.832	0.723	More	0.855	Less	− BMA, α(1–3)-MAN, α(1–6)-MAN‡
5uqy	3.60	5	564B	0.809	0.747	Less	0.785	Less	− BMA, α(1–3)-MAN, α(1–6)-MAN
5utf	3.50	6	88G	0.723	0.372	Less	0.429	Less	− BMA +
5x2p	2.60	3	133B	0.810	0.685	Match	0.760	Less	− BMA
5v2a	4.65	3	28H	0.720	0.605	Match	0.704	More	+ implausible α(1–3)-MAN
5v4e	3.22	5	297A	0.842	0.784	Match	0.754	Less	− 5′ NAG
5v7j	2.91	6	156G	0.712	0.618	Less	—	—	No build§
5vaa	1.55	6	297A	0.881	0.777	Match	0.822	Less	− 5 NAG
5vgj	3.45	5	156G	0.838	0.796	Match	0.726	Less	− BMA +
5vh5	1.75	6	300A	0.787	0.718	Match	0.720	Match	
5vk2	3.20	3	79A	0.794	0.702	Match	0.699	Match	
5nuz	1.85	5	178C	0.774	0.676	Match	0.708	Less	− 4′ α(1–6)-MAN, 5′ α(1–3)-MAN
5nxb	4.60	3	542A	0.741	0.738	Match	0.660	More	+ 4′ MAN
5o32	4.20	4	85A	0.822	0.660	Match	0.747	Less	− BMA, α(1–3)-MAN, α(1–6)-MAN
5vtq	2.95	3	165A	0.802	0.785	Match	0.780	Match	

† Removes CAVEAT A 403 FUC Wrong Chirality C1.

‡ Wispy density for BMA.

§ Poor density for N-linked NAG.
